# Combined transapical transcatheter aortic valve implantation and premature ventricular contraction ablation in a high-risk cardiomyopathy patient: a case report

**DOI:** 10.1093/ehjcr/ytaf441

**Published:** 2025-09-06

**Authors:** Yu-Bin Li, Yang Wu, Chen Su, Zhong-Kai Wu, Li-Chun Wang

**Affiliations:** Department of Cardiology, The First Affiliated Hospital, Sun Yat-Sen University, No. 58 Zhongshan Road 2, 510000 Guangzhou, China; NHC Key Laboratory of Assisted Circulation, No. 58 Zhongshan Road 2, 510000 Guangzhou, China; Department of Cardiology, People’s Hospital of Deqing County, No. 2 Rencuixiang Road, 526000 Zhaoqing, China; Department of Cardiology, The First Affiliated Hospital, Sun Yat-Sen University, No. 58 Zhongshan Road 2, 510000 Guangzhou, China; NHC Key Laboratory of Assisted Circulation, No. 58 Zhongshan Road 2, 510000 Guangzhou, China; Department of Cardiology, The First Affiliated Hospital, Sun Yat-Sen University, No. 58 Zhongshan Road 2, 510000 Guangzhou, China; NHC Key Laboratory of Assisted Circulation, No. 58 Zhongshan Road 2, 510000 Guangzhou, China; Department of Cardiac Surgery, First Affiliated Hospital of Sun Yat-sen University, No. 58 Zhongshan Road 2, 510000 Guangzhou, China; Department of Cardiology, The First Affiliated Hospital, Sun Yat-Sen University, No. 58 Zhongshan Road 2, 510000 Guangzhou, China; NHC Key Laboratory of Assisted Circulation, No. 58 Zhongshan Road 2, 510000 Guangzhou, China

**Keywords:** Transapical transcatheter aortic valve implantation, Premature ventricular contractions, Radiofrequency ablation, Cardiomyopathy, Case report

## Abstract

**Background:**

Frequent premature ventricular contractions (PVCs) and valvular dysfunction are established contributors to worsening heart failure.

**Case summary:**

We present a 67-year-old male with dilated cardiomyopathy, severe aortic regurgitation, and unifocal left ventricle-originated PVCs (37.8% burden) refractory to guideline-directed medical therapy and cardiac devices. Due to high surgical risk, a combined transapical transcatheter aortic valve implantation and PVC ablation was performed. Postoperatively, PVCs were eliminated, left ventricular ejection fraction improved from 35% to 55%, and cardiac dimensions normalized significantly.

**Discussion:**

This case highlights the feasibility of a single apical approach for addressing both valvular and arrhythmic pathologies in high-risk patients, offering a novel strategy to mitigate heart failure progression.

Learning points

- Combined transapical treatment of severe aortic regurgitation and ventricular arrhythmias is feasible in high-risk cardiomyopathy, accelerating reverse remodelling by simultaneously eliminating volume overload and arrhythmia triggers in one procedure.
- Targeted correction of primary aortic regurgitation and premature ventricular contractions may lead to regression of secondary mitral regurgitation without direct intervention, highlighting the importance of addressing underlying ventricular drivers before complex multivalve repair.

## Introduction

Valvular heart disease and ventricular arrhythmias are frequent comorbidities in patients with advanced cardiomyopathy, often synergistically contributing to heart failure progression.^1,2^ Severe aortic regurgitation causes chronic volume overload, leading to left ventricular dilation and dysfunction, while frequent premature ventricular contractions (PVCs) worsen mechanical dyssynchrony and arrhythmia-driven remodelling. Currently, in clinical practice, staged intervention is commonly used, yet isolated treatment of either condition may inadequately address the complex pathophysiology. Transcatheter aortic valve implantation (TAVI) offers a less invasive option for high-risk patients, and PVC ablation can resolve refractory arrhythmias. However, sequential procedures increase risks and recovery time. This case presents a transapical hybrid approach, combining transapical transcatheter aortic valve implantation (TA-TAVI) and left ventricle (LV) originated PVC ablation through a single access site to simultaneously correct valvular dysfunction and eliminate arrhythmic triggers. By integrating structural and electrophysiological therapies, this strategy disrupts the cycle of volume overload and arrhythmia-induced cardiomyopathy, providing a tailored solution for high-risk patients.

## Summary figure

**Figure:**
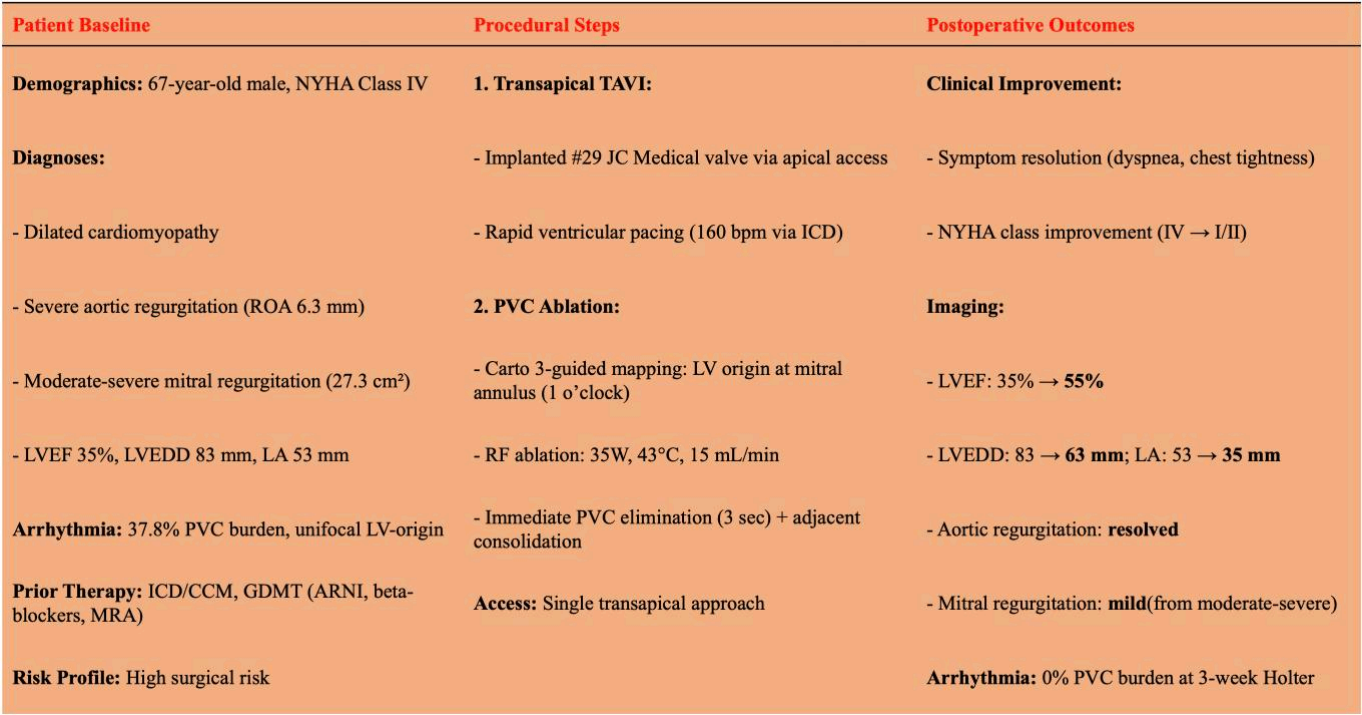


## Case presentation

**Figure 1:**
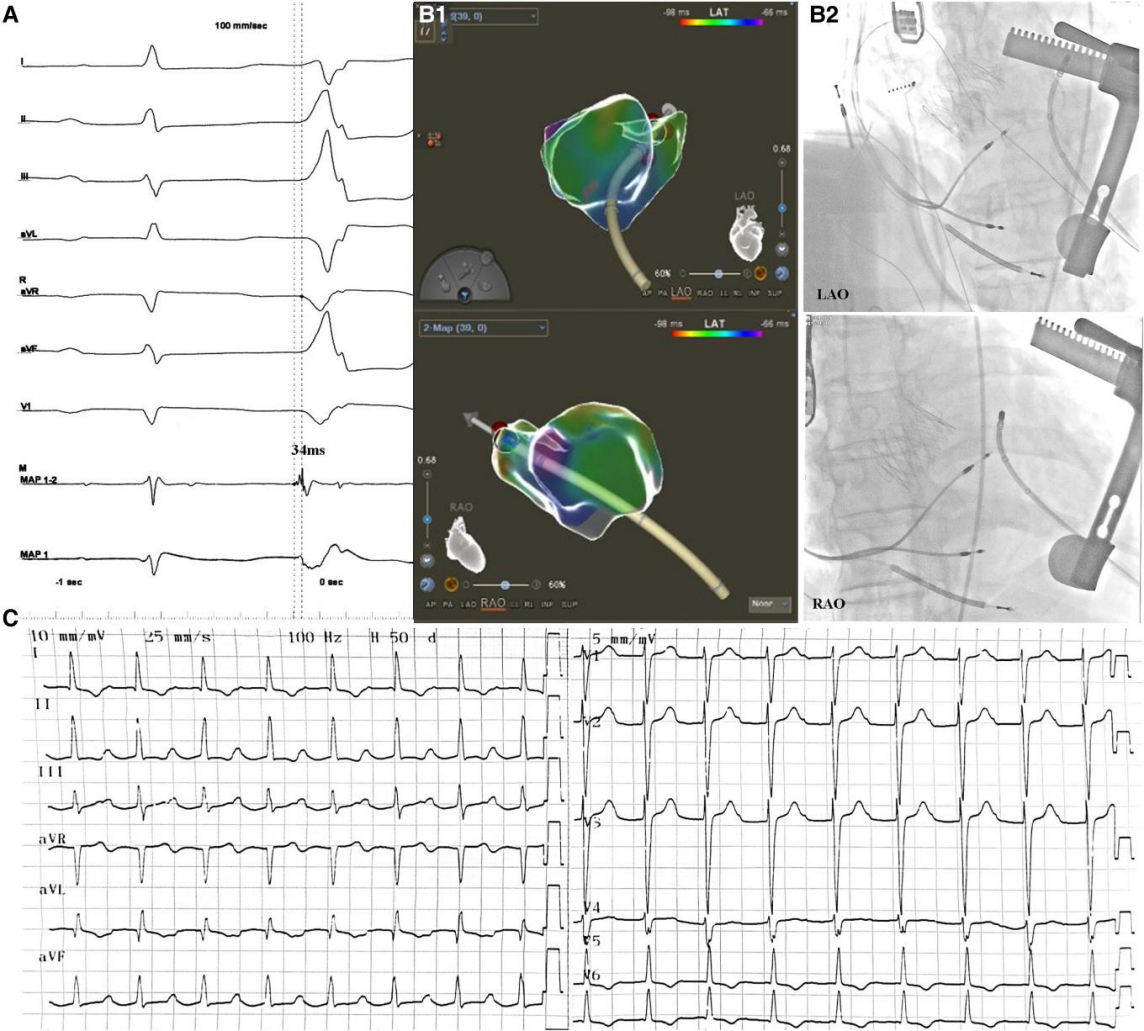
Preoperative electrocardiogram. Sinus rhythm with concomitant first-degree atrioventricular block and frequent ventricular bigeminy. The morphology of the premature ventricular contractions (PVCs) displays an R-wave pattern in precordial lead V1 and Rs or RS patterns in leads V2–V6, suggesting a left ventricular origin.

A 67-year-old male with a 4-year history of chronic heart failure (NYHA Class IV), dilated cardiomyopathy, and severe aortic/mitral regurgitation was admitted for recurrent chest tightness and dyspnoea. Previous interventions included implantable cardioverter-defibrillator/cardiac contractility modulation (ICD/CCM) implantation and optimal pharmacotherapy (beta-blockers, angiotensin receptor-neprilysin inhibitor, mineralocorticoid receptor antagonist, diuretics). Electrocardiogram indicated bigeminy of unifocal PVCs with left ventricular origin (*Figure 1*). Holter monitoring revealed 37.8% PVCs burden, predominantly unifocal, alongside ventricular bigeminy/trigeminy and short runs of non-sustained ventricular tachycardia. Echocardiography confirmed severe LV and left atrium (LA) dilation (LVEDD 83 mm, LA 53 mm), reduced left ventricular ejection fraction (LVEF 35%), severe aortic regurgitation (regurgitant orifice 6.3 mm) and moderate–severe mitral regurgitation (regurgitant area 27.3 cm^2^).

### Intervention

**Figure 2:**
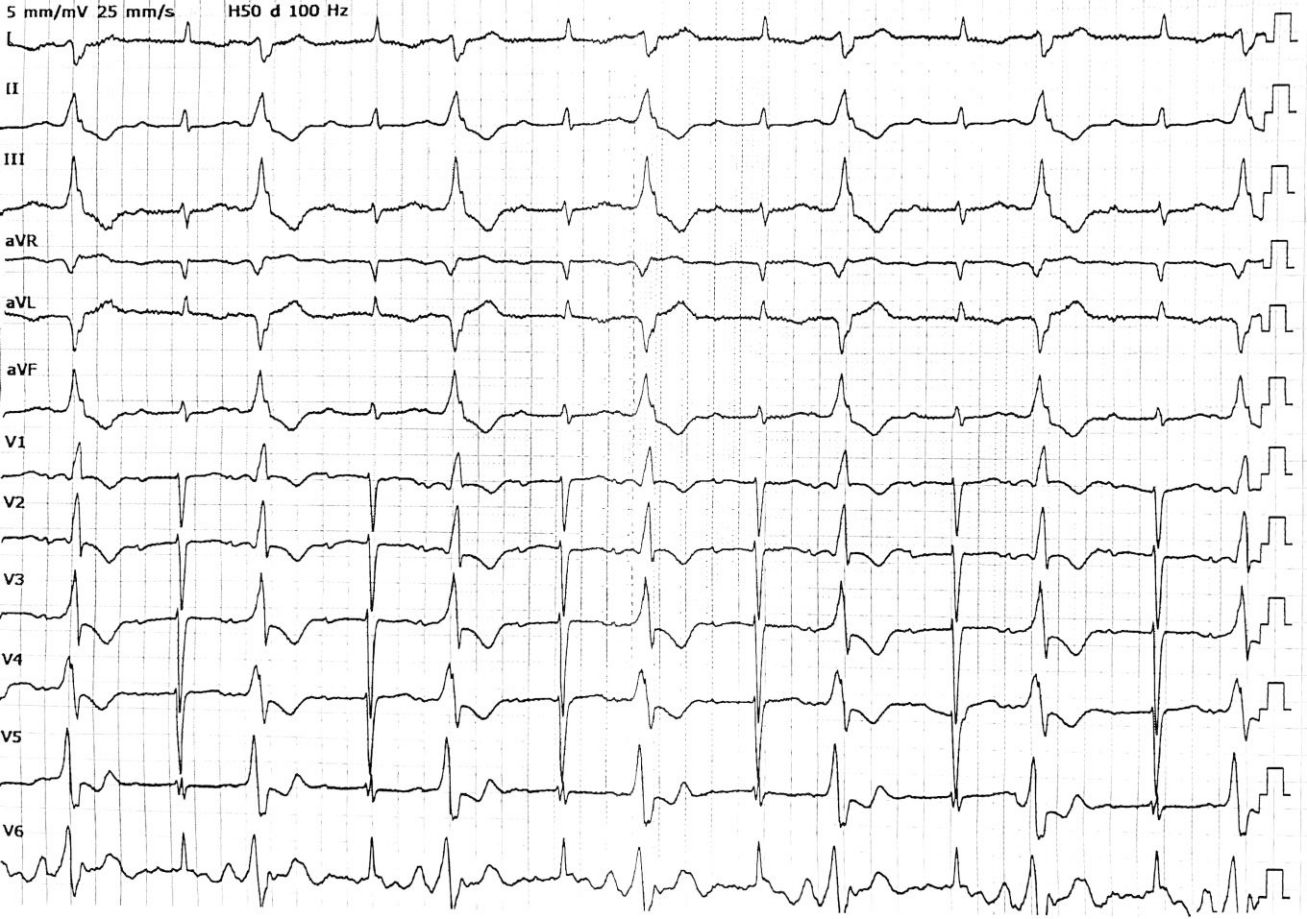
The target of the ablation. (*A*) The electrogram characteristics of the origin of PVC. The earliest activation site precedes the surface electrocardiogram by 34 ms, and atrial potential could be recorded at the ablation catheter (MAP1-2). (*B*) The three-dimension imaging (B1) and X-ray presentation (B2) of the ablative site. (*C*) The postoperative electrocardiogram. RAO, right anterior oblique; LAO, left anterior oblique.

The procedure was performed in a hybrid operating room under general anaesthesia. A #29 transcatheter aortic bioprosthetic valve (JC Medical, Suzhou, PRC) was implanted via transapical access. During the deployment of the valve, rapid ventricular pacing (160 b.p.m.) was achieved through the ICD. Subsequently, an ablation catheter (Thermocool Smarttouch SF, Johnson & Johnson, NJ, USA) was introduced to LV through the same apical access using a short sheath, The PVC origin was mapped using Carto 3 system (Biosense Webster, CA, USA) and identified at the 1 o’clock position of the mitral annulus, and the earliest activation preceded the surface electrocardiogram by 34 ms. Radiofrequency ablation (contact force 8–12 g, 35W, 43°C, 15 mL/min) eliminated PVCs within 3 s, followed by consolidation at adjacent sites (*Figure 2*).

### Outcome

Postoperatively, symptoms such as dyspnoea improved gradually. Three-week follow-up Holter monitoring confirmed complete PVC suppression. Echocardiography demonstrated significant reverse remodelling: LVEDD decreased to 63 mm, LA to 35 mm, and LVEF improved to 55%. Aortic regurgitation resolved completely, and mitral regurgitation improved to mild severity.

## Discussion

Heart failure is one of the ultimate destinations of various heart diseases.^3^ This case demonstrates the synergistic interplay between structural valvular abnormalities and arrhythmic burden in the progression of advanced heart failure, as well as the therapeutic potential of a combined transapical approach to address both pathologies simultaneously.

Structural valvular dysfunction and high-burden PVCs acted synergistically to drive heart failure progression. Severe aortic and mitral regurgitation imposed chronic volume overload, leading to left ventricular dilatation and impaired systolic function. Concurrently, the excessive PVC burden exacerbated myocardial strain through ineffective contractions and arrhythmia-mediated remodelling.^2^ Isolated treatment of either pathology would likely fail to halt disease progression. For instance, correcting aortic regurgitation alone might transiently reduce volume overload, but persistent high-burden PVCs would continue to impair ventricular synchrony and promote arrhythmia-induced cardiomyopathy. Conversely, ablating PVCs without addressing valvular pathology would leave the ventricle vulnerable to ongoing volume stress. Thus, simultaneous intervention was critical to achieve comprehensive reverse remodelling, as evidenced by post-procedural rapid recovery of cardiac dimensions (LVEDD 63 mm) and functional recovery (LVEF 55%). In this case, the rapid recovery of cardiac function and structure suggests that aortic regurgitation and frequent PVCs are the primary causes of the patient’s chronic cardiac insufficiency. Therefore, this case underscores the necessity of a holistic approach in managing multifactorial heart failure aetiologies.

The transapical hybrid approach offered unique advantages in this high-risk patient by enabling simultaneous TA-TAVI and PVC ablation through a single access site. Traditional staged interventions for such patients carry inherent risks. Performing catheter ablation prior to TAVI would require navigating a severely regurgitant aortic valve, increasing procedural complexity and haemodynamic instability; this risk is particularly heightened and potentially prohibitive in patients with such impaired ventricular function, as the procedure may further exacerbate heart failure or precipitate acute intraprocedural decompensation. Conversely, retrograde ablation post-TAVI risks mechanical interference with the prosthetic valve or suboptimal mapping due to restricted catheter mobility. By utilizing a transapical route, both interventions were performed efficiently without compromising the integrity of the newly implanted valve. The apical access allowed direct entry into the LV for precise ablation, avoiding traversal of the aortic valve entirely. This approach not only minimized procedural invasiveness but also capitalized on synergistic benefits: TAVI immediately alleviated volume overload, stabilizing the substrate for ablation, while PVC elimination prevented further arrhythmia-mediated remodelling. To our knowledge, this is the first reported case of combining transapical TA-TAVI and PVC ablation in one procedure. The success of this dual intervention highlights the feasibility of integrating structural and electrophysiological therapies through a unified access strategy, particularly in high-risk patients deemed unsuitable for multiple procedures.

Notably, severe mitral regurgitation (27.3 cm²) improved to mild severity without direct intervention. This highlights the secondary nature of mitral regurgitation in this case, driven primarily by left ventricular dilatation and dyssynchrony. Post-TAVI and PVC ablation, the reduction in LVEDD (83→63 mm) alleviated annular dilation, while PVC abolition also restored coordinated ventricular contraction. These changes enhanced mitral leaflet coaptation, consistent with the concept that functional mitral regurgitation may regress with ventricular reverse remodelling. This observation challenges the necessity of concurrent mitral intervention in select patients.

## Conclusion

The transapical hybrid strategy represents a paradigm shift in managing high-risk patients with combined valvular and arrhythmic pathologies. By concurrently addressing aortic regurgitation and PVCs, this approach disrupts the self-perpetuating cycle of heart failure, facilitating rapid reverse remodelling. It underscores the importance of integrative pathophysiology-driven interventions in complex cardiomyopathy.

## Data Availability

The data was obtained in this article or through the corresponding author.

## Lead author biography

Dr Li-Chun Wang is a distinguished Chief Physician in the Department of Cardiovascular Medicine at the First Affiliated Hospital of Sun Yat-sen University. With over 20 years of expertise in cardiac pacing and electrophysiology, he specializes in the diagnosis and treatment of arrhythmias, syncope, genetic heart diseases, cardiomyopathies, and heart failure. Dr Wang has published over 20 clinical research papers in renowned journals and authored two books. He is a national training instructor for arrhythmia intervention and a CDQI Level 1 Atrial Fibrillation Operator (Excellence Level).

## Notes

**Consent:** The authors confirm that written consent for submission and publication of this manuscript including the images and associated text has been obtained from the patient in line with the COPE guidelines.

**Funding:** None declared.
